# Clinical characteristics of diabetes patients complicated with COVID-19

**DOI:** 10.1097/MD.0000000000039427

**Published:** 2024-11-08

**Authors:** Jie Chen, Haixin Chen, Tingting Chen

**Affiliations:** a Endocrine Department, The First Affiliated Hospital of Zhejiang Chinese Medical University (Zhejiang Provincial Hospital of Chinese Medicine), Hangzhou, China; b The First Clinical Medical College, Zhejiang Chinese Medical University, Hangzhou, China; c Zhejiang Key Laboratory of Integrative Chinese and Western Medicine for Diagnosis and Treatment of Circulatory Diseases, Zhejiang Hospital (Affiliated Zhejiang Hospital, Zhejiang University School of Medicine), Hangzhou, Zhejiang, China.

**Keywords:** coronavirus disease 2019, diabetes, glomerular filtration rate, infection indicators

## Abstract

Patients with both diabetes and coronavirus disease 2019 (COVID-19) are more likely to experience negative outcomes. This study aimed to identify the risk factors associated with these adverse outcomes that can assist clinicians in implementing suitable treatment strategies to minimize the occurrence of severe complications. A total of 92 patients with diabetes and COVID-19 in the Endocrine Department of Zhejiang Provincial Hospital of Chinese Medicine from December 2022 to February 2023 were enrolled and divided into the recovered group and the transfer to the intensive care unit (ICU) or death group. The clinical characteristics and infection indicators were compared between the 2 groups. Additionally, the patients were further divided into the normal group and the reduction group based on their glomerular filtration rate (GFR), and their clinical characteristics and infection indicators were also analyzed. Compared with the GFR normal group, the GFR reduction group exhibited worse outcomes, higher COVID-19 severity, a higher proportion of mechanical ventilation, and a longer hospital stay. However, there were no significant differences in leukocyte, lymphocyte, and neutrophil counts between these 2 groups. Compared with the recovered group, the transfer to ICU or death group demonstrated an increase in leukocytes and neutrophils, while lymphocytes decreased (*P* < .05). The C-reactive protein, procalcitonin, interleukin-6, and serum amyloid A levels in the transfer to ICU or death group were significantly higher than those in the Recovered group (*P* < .05). In addition, C-reactive protein, procalcitonin, and serum amyloid A levels in the GFR reduction group were significantly higher than those in the normal group (*P* < .05), while interleukin-6 levels were only slightly higher (*P* > .05). In clinical treatment, it is necessary to monitor infection indicators and GFR closely and intervene in time to reduce the occurrence of adverse events.

## 1. Introduction

Diabetes is a highly prevalent endocrine disease that affects more than 400 million people worldwide.^[1]^ This disease can cause absolute or relative insulin deficiency, limit the sensitivity of target tissue cells to insulin, and cause a series of metabolic and electrolyte disturbances.^[2]^ Severe hyperglycemia can lead to diabetic ketoacidosis and hypersomnia coma. Long-term illness can also lead to vascular and neurological disorders, which not only influence the quality of life but also shorten their lifespan. Syndrome coronavirus 2 (SARS-CoV-2) has caused a global pandemic with more than 600 million infected and 6.5 million fatalities, and this coronavirus can cause severe viral respiratory disease known as coronavirus disease 2019 (COVID-19).^[3]^ The relationship between diabetes and COVID-19 appears bidirectional.^[4]^ Diabetes patients infected with SARS-CoV-2 are prone to severe pneumonia, multiple organ damage, and even death.^[5,6]^ COVID-19 affects glycemic control, induces diabetic ketoacidosis, and increases the risk of emergency complications.^[7]^ COVID-19 not only exacerbates existing cases of diabetes but may also lead to newly diagnosed cases of diabetes in previously healthy individuals.^[8]^ Several studies have shown that individuals with diabetes who also contract COVID-19 experience more severe symptoms.^[9]^

Diabetic nephropathy is a chronic kidney disease caused by diabetes and is considered one of the most significant microvascular complications.^[10]^ Patients typically present with proteinuria, hypertension, and edema, which can progress to end-stage renal disease.^[11]^ Renal function examination revealed a decrease in the glomerular filtration rate (GFR).^[12]^ Several studies have indicated that individuals with diabetes and chronic renal injury are at a higher risk of developing severe COVID-19-related disease and experiencing higher mortality rates.^[13]^ In our study, we analyzed the clinical characteristics and laboratory indicators of hospitalized diabetes patients with COVID-19 to determine whether a reduction in GFR could be a potential factor contributing to adverse outcomes.

## 2. Materials and methods

### 2.1. Patients and controls

A total of 92 patients with diabetes and COVID-19 in the Endocrine Department of Zhejiang Provincial Hospital of Chinese Medicine, Hangzhou, Zhejiang, China, were enrolled between December 2022 and February 2023. Among them, 67 cases were successfully treated and were discharged from the hospital, defined as the recovered group, while 25 cases deteriorated and were transferred to the intensive care unit (ICU) or died during hospitalization, defined as the transfer to ICU or death group (Table 1). Compared with the recovered group, the transfer to ICU or death group showed a more severe COVID-19 grade, a higher mechanical ventilation rate, and a poorer GFR grade (*P* < .05). However, sex, age, blood pressure, blood fat, length of hospital stay, and urinary ketone levels did not differ significantly between the groups.

**Table 1:** Clinical features of diabetes complicated with COVID-19.

Characteristics	Recovered	Transfer to ICU or death	*P* value
n	67	25	
Sex, n (%)			.377
Male	36 (39.1%)	16 (17.4%)	
Female	31 (33.7%)	9 (9.8%)	
Age (yr), median (IQR)	76 (69, 84.5)	83 (73, 89)	.066
COVID-19 grade, n (%)			<.001
Light	9 (13.43%)	0 (0.00%)	
Medium	20 (29.85%)	0 (0.00%)	
Severe	38 (56.72%)	2 (5.43%)	
Critical	0 (0.00%)	23 (92.00%)	
Blood pressure, n (%)			.952
Hypertension	46 (68.66%)	17 (68.00%)	
Normotensive	21 (31.34%)	8 (32.00%)	
Blood fat, n (%)			.102
Hyperlipidemia	14 (20.90%)	1 (4.00%)	
Normolipidemic	53 (79.10%)	24 (96.00%)	
Mechanical ventilation, n (%)			<.001
No	67 (100.00%)	9 (36.00%)	
Yes	0 (0.00%)	16 (64.00%)	
Hospital stay (d), median (IQR)	12 (9, 17)	12 (8, 21)	.979
GFR, n (%)			.004
CKD 1	35 (52.24%)	5 (20.00%)	
CKD 2	12 (17.91%)	6 (24.00%)	
CKD 3a	10 (14.93%)	2 (9.00%)	
CKD 3b	2 (2.99%)	3 (12.00%)	
CKD 4	2 (2.99%)	6 (24.00%)	
CKD 5	6 (8.96%)	3 (12.00%)	
U-KET, n (%)			.476
Positive	10 (14.93%)	6 (24.00%)	
Negative	57 (85.07%)	19 (76.00%)	

CKD = chronic kidney disease, GFR = glomerular filtration rate, ICU = intensive care unit, IQR = interquartile range, U-KET = urinary ketone.

The clinical classifications of COVID-19 are divided into 4 types according to clinical symptoms, indicators, and imaging findings: mild, common, severe, and critical. Mild: mild clinical symptoms and no sign of pneumonia on imageological examination; common: mild clinical symptoms, with visible signs of pneumonia on imaging; severe: respiratory distress may occur, respiratory frequency ≥ 30/min, oxygen saturation ≤ 93%, ratio of partial pressure of arterial oxygen to fractional concentration of oxygen in inspired air [PaO_2_/FiO_2_] ≤ 300, or lung infiltrates > 50% within 24-48 hours; and critical: occurrence of respiratory failure and need mechanical ventilation, presence of shock and other organ failure, and need ICU monitoring treatment.

According to the international standard for chronic kidney disease (CKD), GFR is divided into 5 stages^[14]^: CKD 1, GFR ≥ 90 mL/(min × 173 m^2^); CKD 2, 90 mL/(min × 173 m^2^) > GFR ≥ 60 mL/(min × 173 m^2^); CKD 3a, 59 mL/(min × 173 m^2^) > GFR ≥ 45 mL/(min × 173 m^2^); CKD 3b, 30 mL/(min × 173 m^2^) > GFR ≥ 44 mL/(min × 173 m^2^); CKD 4, 15 mL/(min × 173 m^2^) > GFR ≥ 29 mL/(min × 173 m^2^); and CKD 5, GFR ≤ 15 mL/(min × 173 m^2^).

The diagnostic criteria for diabetes were as follows: typical symptoms, such as polyuria, polydipsia, weight loss, and fatigue; and randomized glucose ≥ 11.1 mmol/L or fasting blood glucose ≥ 7.0 mmol/L or 2-hour glucose ≥ 11.1 mmol/L.

### 2.2. White blood cell differential counts

Peripheral blood samples were collected in ethylenediaminetetraacetic acid K2 collection tubes to determine the differential white blood cell (WBC) counts. Differential WBC counts were determined using multidimensional analysis techniques of laser scattering combined with nucleic acid fluorescence staining, and C-reactive protein (CRP) detection was performed using latex-enhanced immune scattering turbidimetry. The instruments and reagents were manufactured by the Mindray Company (China).

### 2.3. SAA, PCT, and IL-6 detection

Serum amyloid A (SAA) was generated by Antu Biotech (China) by latex immunoturbidimetry. The instrument used was i2000 (Abbott Laboratories). interleukin-6 (IL-6) and procalcitonin (PCT) were measured using the electrochemical luminescence method, and the instrument used was e601. Both the instruments and reagents were purchased from Roche (Basel, Switzerland).

### 2.4. GFR detection

GFR calculation formula: GFR (male) = (140-Age) × weight × 1.23/blood creatinine. GFR (female) = (140-Age) × weight × 1.03/blood creatinine. Creatinine was evaluated by the creatine oxidase method using C16000 (Abbott Laboratories), and the reagent was purchased from Zhong Sheng Bei Kong Biotechnology Co., Ltd. (China).

### 2.5. Fasting blood glucose and hemoglobin A1c detection

After a fasting period of 8 to 12 hours, 3 mL of venous blood samples were collected to measure fasting blood sugar. The collected blood was then centrifuged at 3000 g/min for 5 minutes. The separated serum was utilized to detect blood glucose levels using a fully automated biochemical analyzer (Abbott C16000). Peripheral blood was collected in ethylenediaminetetraacetic acid K2 collection tubes for hemoglobin A1c (HbA1c) detection. HbA1c was detected using a glycated hemoglobin analyzer (Premier Hb9210).

### 2.6. Statistical analysis

R software (Foundation for Statistical Computing 2020) version 4.2.1 and R packages (ggplot2 [3.3.6], stats [4.2.1], and car) were used for statistical analysis. Statistical significance was set at *P* < .05. If the numerical variables had a normal distribution and homogeneity of variance, a *t* test was used for comparisons between the 2 groups, and a 1-way ANOVA was used for comparisons among the 3 groups. If the numerical variable satisfied the normal distribution but did not satisfy the variance homogeneity test, Welch *t* test was applied for comparisons between 2 groups, and Welch 1-way ANOVA was used for comparisons among the 3 groups.

## 3. Results

### 3.1. The course and outcome of diabetes combined with COVID-19

An analysis of the clinical data from the enrolled patients revealed a significant difference in GFR between the recovered and the transfer to ICU or death groups. Therefore, we assessed fasting blood glucose, HbA1c, the severity, course, and outcomes of COVID-19 in patients with diabetes at different GFR stages. We defined CKD1 (GFR ≥ 90 min × 173 m^2^) as the normal group and CKD2-5 (GFR < 90 min × 173 m^2^) as the reduction group. Compared with the normal group, the reduction group had worse outcomes, a higher severity of COVID-19, a higher proportion of mechanical ventilation, and longer hospital stays. However, there was no significant difference in fasting blood glucose and HbA1c between the 2 groups (Table 2).

**Table 2:** The course and outcome of diabetes combined with COVID-19.

Characteristics	Normal (n = 40)	Reduction (n = 52)	*P* value
Fasting blood glucose (mmol/L), median (IQR)	9.31 (7.35, 12.503)	11.14 (6.95, 14.932)	.272
HbA1c (%), median (IQR)	8.10 (7.10, 10.30)	7.70 (6.85, 8.70)	.294
Overcome, n (%)			.006
Recovered	35 (87.50%)	32 (61.54%)	
Transfer to ICU or death	5 (12.50%)	20 (38.46%)	
COVID-19 grade, n (%)			.005
Critical	5 (12.50%)	18 (34.61%)	
Severe	16 (40.00%)	24 (46.15%)	
Medium	15 (37.50%)	5 (9.62%)	
Light	4 (10.00%)	5 (9.62%)	
Mechanical ventilation, n (%)			.028
No	37 (92.50%)	39 (75.00%)	
Yes	3 (7.50%)	13 (25.00%)	
Hospital stay (d), median (IQR)	10.00 (6.75, 16.00)	13.50 (9.75, 19.00)	.027

HbA1c = hemoglobin A1c, ICU = intensive care unit, IQR = interquartile range.

### 3.2. Insulin and oral hypoglycemic drug use in the GFR normal and reduction groups

The use of hypoglycemic drugs in patients with normal and reduced GFR was analyzed (Supplemental Digital Content, http://links.lww.com/MD/N886), and the results showed that the proportion of insulin usage in the GFR reduction group was higher than that in the normal group, while the proportion of oral hypoglycemic drug usage was lower than that in the normal group (*P* < .05; Table 3).

**Table 3:** Insulin and oral hypoglycemic drug use in the GFR normal and reduction groups.

Characteristics	Normal	Reduction	*P* value
n	40	52	
Insulin usage, n (%)			.025
Yes	21 (52.5%)	39 (75.0%)	
No	19 (47.5%)	13 (25.0%)	
Oral hypoglycemic drug, n (%)			.001
No	16 (40.0%)	38 (73.1%)	
Yes	24 (60.0%)	14 (26.9%)	
Category of hypoglycemic drugs, n (%)			.001
No	16 (40.0%)	38 (73.1%)	
Biguanide	10 (25.0%)	2 (3.8%)	
A-glycosidase inhibitor	9 (22.5%)	2 (3.8%)	
DPP-4 inhibitor	3 (7.5%)	1 (1.9%)	
SGLT2 inhibitor	1 (2.5%)	2 (3.8%)	
Glinides	1 (2.5%)	4 (7.7%)	
Thiazolidinediones	0 (0.0%)	1 (1.9%)	
Sulfonylureas	0 (0.0%)	2 (3.8%)	

DPP-4 = dipeptidyl peptidase-4, GFR = glomerular filtration rate, SGLT2 = sodium-glucose transport protein 2.

### 3.3. WBC differential count

**Figure 1.**
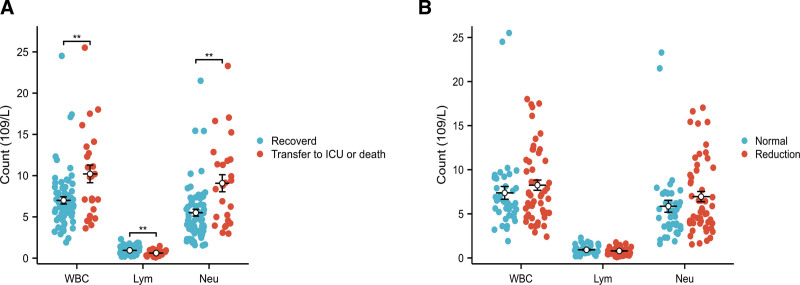
WBC differential count in diabetic. (A) Comparative analysis of WBC differential count between transfer to ICU or death group and recovered group. (B) Comparative analysis of WBC differential count between reduction group and normal group. ICU = intensive care unit, Lym = lymphocytes, Neu = neutrophils, WBC = white blood cells. **P < .01.

The median (interquartile range [IQR]) WBC, lymphocyte, and neutrophil counts in the transfer to ICU or death group were 9.91 (5.91, 12.71) × 10^9^/L, 0.56 (0.45, 0.75) × 10^9^/L, and 8.79 (4.54, 11.80) × 10^9^/L, respectively. The median (IQR) of leukocytes, lymphocytes, and neutrophils in recovered group were 6.21 (5.11, 8.01) × 10^9^/L, 0.85 (0.62, 1.22) × 10^9^/L, and 4.69 (3.49, 6.65) × 10^9^/L, respectively. Compared with the recovered group, the transfer to ICU or death group showed an increase in leukocytes and neutrophils, while lymphocytes decreased (*P* < .05; Fig. 1A).

The median (IQR) of WBC, lymphocytes, and neutrophils in GFR reduction group were 7.11 (5.11, 11.035) × 10^9^/L, 0.72 (0.51, 1.04) × 10^9^/L, and 5.54 (3.95, 9.54) × 10^9^/L, respectively. The median (IQR) of leukocytes, lymphocytes, and neutrophils in the normal group were 6.36 (5.19, 8.34) × 10^9^/L, 0.75 (0.60, 1.16) × 10^9^/L, and 5.17 (3.59, 7.29) × 10^9^/L, respectively. There were no significant differences in leukocyte, lymphocyte, or neutrophil counts between the GFR reduction and the normal groups (Fig. 1B).

### 3.4. Analysis of infection indicators in patients with different outcomes

**Figure 2.**
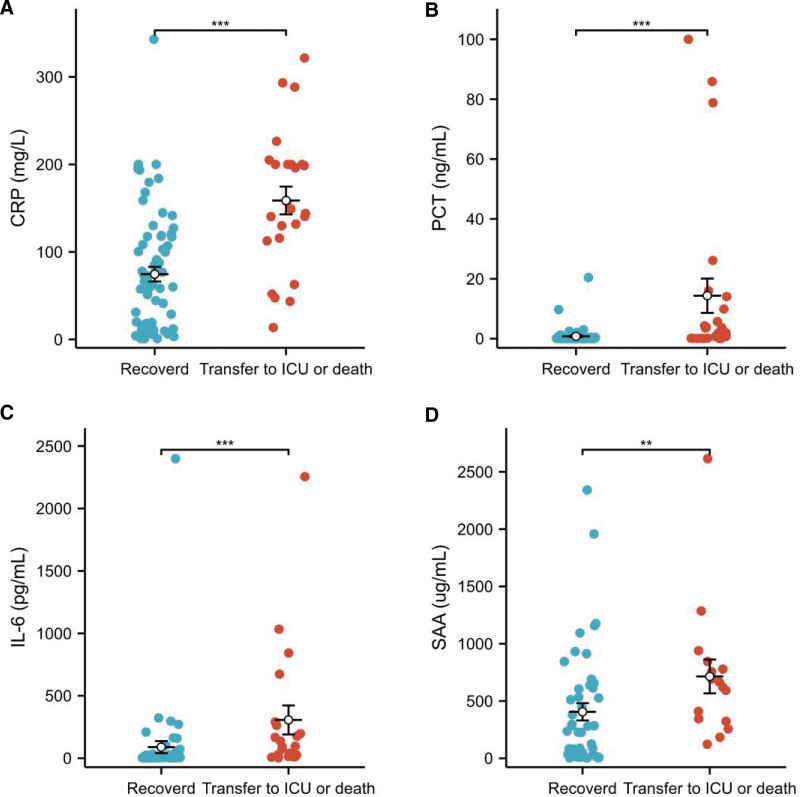
Analysis of infection indicators in patients with different outcomes. (A) Comparative analysis of CRP in patients with different outcomes. (B) Comparative analysis of PCT in patients with different outcomes. (C) Comparative analysis of IL-6 levels in patients with different outcomes. (D) Comparative analysis of SAA among patients with different outcomes. **P < .01; ***P < .001. CRP = C-reactive protein, ICU = intensive care unit, IL-6 = interleukin-6, PCT = procalcitonin, SAA = serum amyloid A.

We assessed routine indicators of infection in the enrolled patients, including CRP, PCT, IL-6, and SAA levels. The median CRP of the transfer to ICU or death group was 148.97 (115.70, 200.00) mg/L, while the median CRP of the recovered group was 62.54 (12.81, 117.43) mg/L. The CRP level of the transfer to ICU or death group was significantly higher than that of the recovered group (*P* < .05; Fig. 2A).

Among the enrolled patients, 6 recovered patients did not undergo PCT testing. The median PCT of the transfer to ICU or death group was 1.94 (0.15, 9.89) ng/mL, while the median PCT of the recovered group was 0.09 (0.06, 0.27) ng/mL. The PCT level in the transfer to ICU or death group was significantly greater than that in the recovered group (*P* < .05; Fig. 2B).

Among the enrolled patients, 50 cases in the recovered group and 21 cases in the transfer to ICU or death group underwent IL-6 testing. The median IL-6 level was 96.33 (26.26, 262.29) pg/mL in the transfer to ICU or death group and 5.69 (3.82, 27.58) pg/mL in the recovered group. The IL-6 levels in the transfer to ICU or death group were remarkably higher than those in the recovered group (*P* < .05; Fig. 2C).

Forty-six patients in the recovered group and 16 cases in the transfer to ICU or death group underwent SAA testing. The median of SAA was 643.05 (339.45, 795.28) ug/mL in the transfer to ICU or death group and 232.15 (32.50, 610.62) pg/mL in the recovered group. The SAA in the transfer to ICU or death group was noticeably higher than that in the recovered group (*P* < .05; Fig. 2D).

### 3.5. Analysis of infection indicators in GFR normal and reduction groups

**Figure 3.**
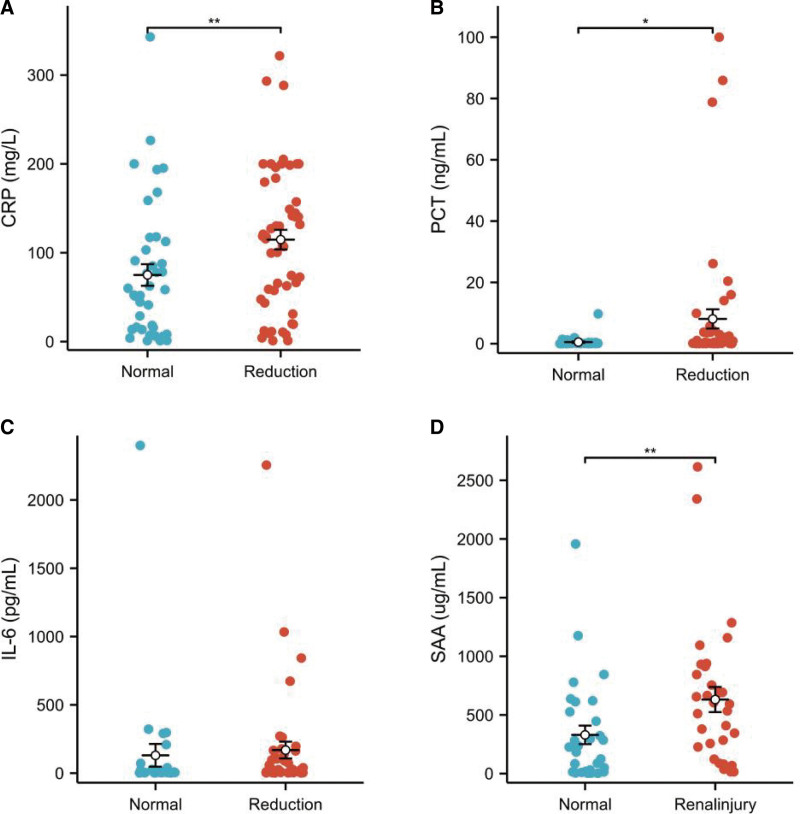
Analysis of infection indicators in GFR normal and reduction groups. (A) Comparative analysis of CRP in GFR normal and reduction groups. (B) Comparative analysis of PCT in GFR normal and reduction groups. (C) Comparative analysis of IL-6 levels in GFR normal and reduction groups. (D) Comparative analysis of SAA in GFR normal and reduction groups. *P < .05; **P < .01. CRP = C-reactive protein, GRF = glomerular filtration rate, IL-6 = interleukin-6, PCT = procalcitonin, SAA = serum amyloid A.

The median CRP level of the GFR reduction group was 118.17 (55.05, 162.87) mg/L, while that of the normal group was 55.40 (13.61, 105.51) mg/L (Fig. 3A).

Forty-seven patients in the GFR reduction group and 37 patients in the normal group underwent PCT testing. The PCT level in the reduction group was 0.21 (0.08, 3.59) ng/mL, while that in the normal group was 0.09 (0.06, 0.26) ng/mL (Fig. 3B).

IL-6 was detected in 42 patients in the GFR reduction group and in 29 patients in the normal group. The IL-6 levels in the reduction group were 26.00 (5.72, 153.80) pg/mL, while those in the normal group were 5.06 (3.91, 26.88) pg/mL (Fig. 3C).

Thirty-two patients in the GFR reduction group and 30 in the normal group underwent SAA testing. The SAA in the reduction group was 563.40 (201.12, 861.28) ug/mL, while that in the normal group was 205.00 (15.50, 506.28) ug/mL (Fig. 3D).

CRP, PCT, and SAA levels were significantly higher in the GFR reduction group than in the normal group (*P* < .05). The IL-6 levels in the GFR reduction group were slightly higher than those in the normal group, but the differences were not significant (Fig. 3).

## 4. Discussion

At the end of 2019, the global pandemic of SARS-CoV-2 resulted in an increase in COVID-19-related mortality. Patients with metabolic diseases have a higher risk of SARS-CoV-2 infection, a higher mortality rate, and are more susceptible to long-term COVID-19.^[15]^ Diabetes is identified as one of the main risk factors for COVID-19.^[16]^ The spike protein of SARS-CoV-2 binds to the ACE2 receptor in liver cells, which stimulates gluconeogenesis and leads to hyperglycemia.^[17,18]^ Additionally, SARS-CoV-2 infection can trigger the secretion of inflammatory factors in the body.^[19]^ The condition worsens when an inflammatory factor storm is triggered, causing damage to multiple organs such as the lungs, kidneys, liver, and heart. Some studies have shown a positive correlation between hyperglycemia and inflammation after SARS-CoV-2 infection.^[20]^ Hyperglycemia and inflammation have a 2-way relationship.^[21]^ In patients with type 2 diabetes, changes in the number and activation status of white blood cells can increase apoptosis and tissue fibrosis, and COVID-19 can further exacerbate insulin resistance, leading to inflammatory stress hyperglycemia.^[19]^ Therefore, we analyzed the clinical data and inflammatory indicators of patients with diabetes who experienced different outcomes during the initial COVID-19 outbreak in our hospital. We discussed the potential causes of adverse outcomes, emphasizing the need for clinicians.

We categorized the enrolled patients into 2 groups: the recovered group and the transfer to ICU or death group. The clinical data of both groups were analyzed. In the transfer to ICU or death group, 92% of patients with COVID-19 were classified as critical, whereas in the recovered group, the severity of COVID-19 was significantly lower, with no critical cases. The transfer to ICU or death group had a significantly higher proportion of mechanical ventilation and a greater decrease in GFR compared to the recovered group. We compared the fasting blood glucose, HbA1c, severity of COVID-19, and prognosis between the GFR reduction group and the normal group. The results showed no significant difference in fasting blood glucose and HbA1c levels between the 2 groups, indicating consistent blood glucose baselines. However, the reduction group exhibited a higher severity of COVID-19, with critical and severe cases being predominant, whereas the normal group mainly had severe and medium cases. In an English cohort study on diabetes patients combined with COVID-19, renal impairment based on the estimated GFR was linked to adverse outcomes in COVID-19 patients with diabetes.^[22]^ If patients with diabetes are not treated promptly, long-term hyperglycemia can have negative effects on renal hemodynamics and metabolic abnormalities, ultimately leading to renal damage. In our analysis, we examined the use of hypoglycemic drugs among diabetes patients categorized by their GFR groups. We found that insulin was the primary choice in the GFR reduction group, whereas the GFR normal group had a significantly higher proportion of oral hypoglycemic drug usage. This difference can be attributed to the fact that common oral hypoglycemic drugs are eliminated from the body through renal clearance. In patients with moderate to severe CKD (CKD3-5), the administration of oral hypoglycemic drugs can lead to drug accumulation and worsen the occurrence of adverse reactions. Therefore, in clinical treatment, patients with concurrent renal insufficiency are more likely to use insulin for blood sugar control. SARS-Cov-2 can target the ACE2 receptor on the surface of the kidney, leading to proteinuria, kidney damage, and even failure.^[23]^ Large viral invasions can have serious consequences, including adverse outcomes and death. Our study revealed that patients with a decreased GFR were significantly more likely to be transferred to the ICU or experience mortality compared to those with a normal GFR. This finding further supports the notion that GFR can be considered a risk factor that influences patient outcomes.

We compared the differential WBC counts and infection indicators in patients with different outcomes. The CRP, PCT, IL-6, and SAA levels in the transfer to ICU or death group were noticeably higher than those in the recovered group. In a study by Haroun et al^[24]^, the CRP, IL-6, and PCT levels of deceased COVID-19 patients were significantly higher than those of survivors. In the “New Coronavirus Pneumonia Diagnosis and Treatment Program (Trial Version 10),” PCT, CRP, IL-6, and other indicators are regarded as early warning indicators for disease deterioration. Some studies have shown that when infected with COVID-19, a CRP threshold value of 50 mg/L can identify severe/critical illness, and higher than 75 mg/L can indicate a high risk of death.^[25]^ High PCT levels are closely associated with mortality.^[26,27]^ IL-6, as a key proinflammatory factor in the body, not only stimulates the activation of CD8^+^ T and B cells and promotes highly specific adaptive immunity but also leads to the activation of matrix metalloproteinases, damages the basement membrane and extracellular matrix, and increases tissue permeability.^[28]^ Severe COVID-19 leads to the excessive production of IL-6 and excessive activation of immunity, and cytokine storms will eventually lead to lung injury and multiple organ failure. As inflammatory factors are closely associated with the outcome of COVID-19 patients and the GFR of patients with adverse outcomes is reduced, we compared the infection indicators of patients with normal GFR and those with reduced GFR. Compared with the GFR normal group, the GFR reduction group showed significant increases in CRP, PCT, and SAA (*P* < .05), while IL-6 slightly increased (*P* > .05). IL-6 levels did not increase significantly compared to other infection indicators, which may be related to missing detection in some samples. Renal injury and inflammatory factors are the main factors that influence the unfavorable outcomes of COVID-19. Renal injury often occurs in patients with diabetes due to poor blood sugar control or delayed treatment. When the damaged kidney is attacked by SARS-Cov-2 again, acute renal injury is more likely to take place. Therefore, we are required to pay close attention to the changes in inflammatory factors and GFR in patients with diabetes and COVID-19 and to control these factors to reduce adverse events.

Our study has certain limitations. First, because the symptoms of ordinary COVID-19 patients are mild, they were not hospitalized to monitor infection indicators. Therefore, we did not include the uninfected individuals in the control group. Second, the number of enrolled patients was insufficient to conduct the univariate and multivariate regression analyses. Finally, the included patients were infected with SARS-Cov-2 for the first time, and the prognosis of patients with multiple infections may differ. The pathogenicity of different strains also varies, leading to differences in patient prognoses.

## 5. Conclusions

Patients with diabetes and COVID-19 are prone to adverse outcomes and susceptible to long-term COVID-19. The proportion of GFR reduction in the transfer to ICU or death group was significantly higher than that in the recovered group. As a common complication of diabetes, the GFR reduction group had worse outcomes, a higher COVID-19 severity, a higher proportion of mechanical ventilation, and longer hospital stay than the normal group. In clinical treatment, we need to pay close attention to the GFR and infection indicators of patients with diabetes complicated by COVID-19 infection and intervene in time to reduce adverse outcomes.

## Author contributions

**Data curation:** Jie Chen, Haixin Chen.

**Funding acquisition:** Jie Chen, Tingting Chen.

**Investigation:** Jie Chen.

**Methodology:** Jie Chen, Haixin Chen.

**Conceptualization:** Haixin Chen.

**Formal analysis:** Haixin Chen.

**Writing – original draft:** Tingting Chen.

**Writing – review & editing:** Tingting Chen.

## Supplementary Material


